# Rectal Neuroendocrine Neoplasms: Why Is There a Global Variation?

**DOI:** 10.1007/s11912-021-01172-1

**Published:** 2022-01-27

**Authors:** Jack Cope, Raj Srirajaskanthan

**Affiliations:** 1 Department of Gastroenterology, Kings College Hospital, London, SE5 9RS UK; 2 Kings Health Partners ENETs Centre of Excellence Neuroendocrine Tumour Unit, Institute of Liver Studies, Kings College Hospital, London, SE5 9RS UK

**Keywords:** Rectal neuroendocrine tumour, Ethnicity, Bowel cancer screening, Polyp, Endoscopy, Registry, Healthcare

## Abstract

**Purpose of Review:**

This review examines the variation in incidence of rectal neuroendocrine tumours across the globe. Rectal neuroendocrine tumours are a common type of gastrointestinal NET with an increasing incidence reported over the last 30 years.

**Recent Findings:**

There have been a number of publications examining the epidemiology of neuroendocrine tumours across the world. These have utilized a variety of different methodologies to examine both incidence of prevalence of NETs. We review the data published and describe any causative factors and findings regarding the epidemiology of rectal NETs.

**Summary:**

Rectal NETs account for 1–2% of all rectal cancers and are commonly diagnosed between 50–60 years of age. Most lesions are identified by chance at colonoscopy, commonly during colon cancer screening procedures, which is reflected in part in the age at diagnosis. Most lesions are small in size, < 10 mm and can be managed with endoscopic resection rather than requiring surgery. The highest incidence is reported in people of Asian ethnicity, with a tenfold increased incidence reported in some series compared with white population. There is also an increased incidence in Black and Hispanic population as identified through the Surveillance, Epidemiology and End Results (SEER) database. Endoscopic assessment of lesions is variable globally. Future work to better understand the cause of ethnic variation and development of comprehensive cancer registries would be helpful.

## Introduction

Rectal neuroendocrine neoplasms (NENs) are increasing in incidence in many parts of the globe [1••, 2•, 3••]. In some countries, such as Korea and Japan, rectal NENs are the most common type of gastroenteropancreatic NENs [4, 5]. This article reviews the current epidemiological data regarding rectal NENs and investigates as to the possible reason for marked variation in incidence of this type of neuroendocrine tumour.

Rectal NENs encompass rectal neuroendocrine tumours (NETs) and rare poorly differentiated neuroendocrine carcinomas (NECs) and mixed neuroendocrine-non neuroendocrine neoplasm (MiNEN). The well-differentiated rectal NETs can be sub-classified by tumour cell type to L-cell rectal NETs which are the most common sub-type and the EC-cell rectal NETs [6••]. The L-cell rectal NETs are commonly small with 75–88% being less than 1 cm in size; on immunohistochemical analysis, they may be chromogranin A (CgA) and CDX-2 negative. However, they can stain positive for glucagon like peptides (GLP-1) and pancreatic polypeptide (PP). Conversely, the EC-cell rectal NETs have immunohistochemical features similar to small bowel NETs and are often CgA and CDX-2 positive [6••]. There is some evidence to suggest a non-L-cell immunophenotype is associated with more aggressive clinical behaviour and worse prognosis. A recent study demonstrated non-L-cell type phenotype with worse prognosis on univariate analysis when studying 10-year survival. If non-L-cell immunophenotype is combined with tumour size > 1 cm then outcomes are significantly worse than other subtype of rectal NET [7]. However, this finding has not been replicated in other studies which demonstrate no significant difference in outcomes whether tumours are L-cell phenotype or not [8].

Most rectal NETs are small and often diagnosed at an early stage of disease. The prognosis of rectal NETs is the best of all GEP-NETs in some series [9, 10]. This is largely due to the high incidence of small rectal NETs that have no evidence of lymphovascular invasion and carry an excellent long-term prognosis [3••, 11]. The 5-year survival rates of rectal NETs are reported at 74–88% from a Norwegian register, SEER database and Chinese centres [5, 12, 13]. Data from studies in China and Korea [14•, 15] have demonstrated over 85% of rectal NETs that are stage 1 or 2 at diagnosis. Therefore, these patients have an excellent long-term prognosis. Most rectal NETs as stated earlier are < 10 mm and the majority are grade 1 tumours. A large series from Korea of 567 patients undergoing endoscopic resection demonstrated 79.9% as grade 1 tumours and the remaining lesions were grade 2 [15], and similar findings from other studies in Japan demonstrate that over 90% of rectal NETS were grade 1 and 62% in a study by Yu et al*.* from China [16].

There has often been a reported correlation with tumour grade and size; however, this is not always the case [10, 17]. Endoscopic resection has often been regarded as a suitable treatment modality for lesions up to 10 mm and even extending to 20 mm [9]. There is increasing data to suggest that lymph node metastasis can occur in lesions < 10 mm and therefore, formal staging assessment should be performed prior to resection of these lesions to exclude lymph node metastasis. A recent study from Inada et al. demonstrated that in ≤ 10-mm lesions, there was 3% lymph node involvement in surgically resected cases [18•]. Other series have demonstrated similar findings with reported lymph node rate involvement in sub-1-cm lesion [19••, 20, 21]. As a result of these findings, it would be reasonable to stage all < 1-cm rectal NETs with MRI at baseline to ensure no nodal involvement. CT can be of limited value in identifying nodal disease in rectal NETs [22]. The ENET guidelines also suggest a role for colonic ultrasound; however, this endoscopic investigation has limited availability. For lesions between 10 and 20 mm, there is a higher risk of nodal involvement and therefore careful pre-operative staging is required to ensure there is no nodal involvement and resection technique will enable an R0 resection to be considered. For lesions > 20 mm, the risk of nodal involvement is over 60% and therefore ENET guidelines recommend surgical oncological resection.

In terms of endoscopic resection technique employed, there is a wide variation both globally and within centres. This is primarily directed by the expertise available at different institutions. The largest published series from Southeast Asia have examined different endoscopic resection techniques using endoscopic mucosal resection (EMR), cap assisted EMR, band assisted EMR, or endoscopic mucosal dissection (ESD) [9, 10, 15, 23]. The consensus is that the standard snare polypectomy is insufficient to enable clear resection margins in the majority of cases. Therefore, recommendation is for a more advanced endoscopic technique using EMR or ESD. Due to variation in endoscopy practice and training in western countries, rectal NETs are frequently incompletely excised at the baseline endoscopy procedure. A multicentre study demonstrated only 18% of French endoscopists suspected a rectal NET at time of index endoscopic procedure. Furthermore, of the 345 cases included in the study, 100 required a second procedure to resect the residual lesion. A R0 resection was only achieved in 17% of those lesions resected by EMR or snare polypectomy [19••]. This is usually due to not identifying the lesion as a rectal neuroendocrine tumour at the initial assessment and commonly misdiagnosing it as a hyperplastic polyp or lipoma and therefore, biopsy of the lesion or undertaking is a conventional snare polypectomy. Due to the lack of awareness of the endoscopic features of rectal NETs and common misdiagnosis of these lesions as hyperplastic polyps, it is possible that under diagnosis is made at endoscopy. Since normal endoscopic practice would not recommend sampling or removal of small hyperplastic polyps. Whilst in the Far East where endoscopic practice and lesion recognition is different, there may be a higher detection of rectal NETs at colonoscopy and so leading to a higher incidence. However, there is no published evidence comparing endoscopic lesion recognition between eastern and western endoscopists to support this hypothesis.

## Epidemiology of Rectal NETS in Europe

Historic data regarding incidence of GEP-NETs in Europe suggested that incidence of rectal NETs was low in European countries [24]. With reported incidence of 1.04 per 100,000 population in the SEER dataset compared with the reported incidence of 0.1–0.3 per 100,000 population in European countries (UK, Austria, Italy, Norway and Sweden) [2•, 25, 26••], analysis of the public health England National Cancer Registry Analysis Service (PHE NCRAS) identified rectal NET incidence of 0.32 per 100,000 population between 2013–2015, with median age of diagnosis 60–64 years old [3••]. This demonstrates a threefold increase in reported incidence in the UK between 2013–2015 compared with 1971–2006 [3••, 25]. This could be related to the advent of colon cancer screening since the average age of diagnosis is that of the bowel cancer screening population. Data from other European countries also confirm a low incidence of rectal NETs. A study examining data from two Swiss cancer registries, Vaud and Neuchatel cancer registry, between 1974 and 2016, identified 4141 cases with NET in a population of approximately 951,514. Total GEP-NET incidence was 4.2 cases per 100,000 population, of which rectal NETs comprised up to 10% of cases. Therefore, rectal NET incidence was 0.42 per 100,000 population, which is similar to that reported in England [3••, 27]. A study of an East German cancer registry noted an increased incidence of 270% between 1976 and 2006, which the investigators attributed to increased endoscopic and radiological investigations. The reported incidence of rectal NETs in 2004–2006 was 0.25 per 100,000 population [28].

## Epidemiology of Rectal NETs in North America

Rectal NETs have a reported increasing incidence in the USA. The SEER database is the most used database. Surveillance, Epidemiology and End Results (SEER) database covers approximately 17% of the US population [2•]. The SEER database from 1975 to 2012 has demonstrated a continual rise in incidence in rectal NETs. An analysis by Lawrence et al. demonstrated rectal NET as the most common type of NET accounting for 17.7% of all NETs [29]. The estimated incidence of rectal NETs is 1.04 cases per 100,000 population [1••]. Based on the SEER database, the overall survival for rectal NETs is reported at 24.6 years [26••]. The SEER database is one of the most comprehensive resources for displaying how ethnic variation can affect NET type, incidence, and survival, which is discussed in further detail later.

A retrospective population-based study using the Ontario cancer registry between 1994 and 2009 noted rectal NETs as the most common type of GEP NET with a reported incidence of 0.74 cases per 100,000 population. This is like the data from the USA. The 5-year survival rate of rectal NET was reported at 73.4% [30].

## Epidemiology of Rectal NETs in Asia

There is limited data from India in terms of incidence of GEP NETs. A study from 6 tertiary care centres, rather than a population or government registry, was performed. Within this publication, they have combined colonic and rectal NETs, with a reported 9% of these comprised of rectal NETs [31]. The series was unable to determine what the population incidence would be; however, as this contributes to only a small percentage of GEP-NETs, it can be postulated the rectal NETs are not the most common type of GEP-NETs and account for a similar percentage of rectal NETs as other European countries.

A recent study from Japan examined data collected from 25 institutions. A total of 416 patients were identified from the centres of which 390 patients had full datasets could be examined. They demonstrated a male preponderance of 61%. The majority of tumours were sub-10 mm, 300/390 = 77% [4]. A 2005 study by Ito demonstrated that 60% of GEP-NETs in Japan are Hindgut in origin [32]. This high incidence of rectal NETs is replicated throughout other countries in East Asia.

A recent study from China identified rectal NETs as the second most common type of GEP-NET accounting for 29.6% of cases, second only to pancreatic NETs accounting for 31.5%. This data from 2001 to 2010 from 25 tertiary referral hospitals and 2010 cases were available for review [33]. Another study from China assessing 168 NETs from 2003 to 2009 from two academic centres reported 58.9% rectal NETs. Of the 99 cases of rectal NETs, interestingly in this study, 40% was < 10 mm in size and the remaining is larger than 1 cm. This is a lower number of small rectal NETs than other studies; however, this could be related to the nature of referrals received by the tertiary centre [5]. This is not from a national cancer registry and therefore will not be as comprehensive.

In Korea, there is a national registry of NETs diagnosed between 2000 and 2009 for 4951 GEP-NET patients. In this series, 48% of patients had rectal NETs [34]. A further study examining data from a single centre of 125 GEP NET patients noted that 79.8% of patients had rectal NETs. The majority of the rectal NETs were grade 1 and in an early stage of disease [35]. A large multicentre study of 652 patients with rectal NETs was diagnosed at 16 centres between 2000 and 2012. As with other studies, there was a slight male predominance with 59.7% of patients being male. As expected, most tumours were less than 10 mm in size in 85% of cases.

Epidemiology of NETs was queried through the Taiwan Cancer Registry between 1996 and 2008 by Tsai et al. [36] [37]. They identified 2187 cases of which 45.1% was rectal NETS. This was the most common type of NET within the population. Interestingly, they noted an increased annual incidence of rectal NETs of 0.31 per 100,000 population between 1996 and 2008. The 5-year overall survival for rectal NETs was excellent at 80.9%. This registry analysis was updated by Chang et al. from the period of 1996 to 2015 [38]. This again demonstrates an increasing incidence of rectal NETs with annual incidence of 1.2 per 100,000 population in 2015. The 10-year overall survival remains very good at 77.5%.

## The Role of Colon Cancer Screening and Endoscopy

Endoscopy and colon cancer screening have been attributed as one of the main causes of elevation in rectal NET incidence in some countries. However, there is a significant variation in the type and approach to colon cancer screening programmes worldwide and therefore may lead to a bias in incidence in some countries. The two main models of colon cancer screening are single step, i.e., invitation to the whole age group to undergo a screening colonoscopy. In the USA, this is offered from the age of 45 and occurs every 5 years or sooner depending on findings. Whilst a number of European countries offer a two-step approach, which uses a stool test, commonly a quantitative faecal immunohistochemical test (qFIT) which measures blood within the stool. There are different thresholds in different countries; however, this test allows those with a positive test to be offered a screening procedure.

A study by Basuroy et al. identified 147 NETs diagnosed following 216,707 screening colonoscopies in the UK. Eighty percent of the NETs identified at colonoscopy were rectal NETs, which gave an incidence of 29 rectal NETs per 100,000 colonoscopies [39]. Of the rectal NETs diagnosed, 85% was stage 1 and 80% was grade 1 on histology. The incidence of rectal NETs in the colonoscopy screening population was much higher than the reported incidence of the PHE NCRAS database of 0.32 per 100,000 [3••]. A study from Poland assessing incidence of rectal NETs from a single Polish BCSP screening centre identified an incidence of 48 per 100,000 colonoscopies [40]. Interestingly in this study, there is a 1-step screening process rather than using a faecal test to identify at-risk individuals.

A Dutch group identified an increasing incidence of colorectal NETs between 2006 and 2016. The initial rise from 2006 to 2011 was from 0.36 per 100,000 population to 0.75 per 100,000 in 2011. It remained at this level until 2016. The majority of lesions were rectal NETs at 76.4%, which would equate to an incidence 0.55 per 100,000 population. They have identified that between 2014 and 2016, 31.9% of colorectal NETs were diagnosed via the screening programme. Of these colorectal NETs, 83.1% was in the rectum. They conclude that the screening programme has directly led to an increasing incidence of rectal NETs. The majority of the lesions detected are at early stage and managed without need for surgery [41].

## Ethnic Variation in USA SEER Data

The SEER database is comprised of 17 cancer registries of the National Cancer Institute. This covers 28% of the US population, approximately 90 million people. It records tumour characteristics as well as individual demographic including race. Yao et al. initially published some of the early data suggesting ethnic variations in type of GEP-NETs. They reported rectal NETs occurred at a markedly higher frequency amongst Asian/Pacific Islander (41%), American Indian/Alaskan Native (32%), and African American (26%) patients than amongst white (12%) patients [2•].

A study examining the SEER database from 1992 to 2008 reported an increase in the detection of rectal carcinoids in the post screening colonoscopy era compared to the pre-colonoscopy screening era. The age of screening was 50 years old and so there is a significant rise in the diagnosis of rectal NET in the 50–59 year group when comparing the pre-colonoscopy screening to post-colonoscopy screening era (390 vs 1379) [42]. This is also seen in the other age groups. Interestingly, the mean age of diagnosis remains the same at 56 in both eras [42]. In this study, people of Asian ethnicity are more likely to be diagnosed with a rectal NET than white ethnicity (*OR* 10.063, 05% *CI*, 8.330–12.157), *p* < 0.001).

A study looking at the incidence and survival amongst Americans in the SEER database identified that black people were more likely than white people to develop GEP-NETs. Furthermore, they reported a higher incidence of a colorectal NET in local, regional and metastatic disease. Therefore suggesting that due to poor access to screening and diagnostic procedures, Black individuals are more likely to present with advanced disease [42, 43]. A similar finding was reported in the access of blacks to colon cancer screening and the high incidence but with advanced disease [44]. There appears to be obstacles in terms of accessing screening and this is due to socio-economic status amongst other issues.

## Limitations in Understanding Global Registries

The data available to understand the incidence of rectal NETs across the globe is very heterogenous. This leads to significant limitations in terms of interpreting the available data. The SEER database covers a population of almost 90 million and provides the best demographic data covering different ethnicities. However, it does have specific limitations and that it does not cover 72% of the US population and historically, coding of tumours was not very accurate. This has improved with time and current versions using the most accurate ICD-09 codes enable accurate data capture. Interestingly, the impact of rectal NETs on colon cancer screening programmes in the US has been reported (impact of rectal NET on rectal cancer screening age 26••). The PHE database in England is also a very detailed database, again limitation was historically present due to coding issues, which appears to have been resolved. Also, the PHE dataset gives full coverage for the whole of England which is very helpful. Studies from the Netherlands again use national registries which enable comprehensive data capture and therefore clear trends in the incidence.

However, a number of Southeast Asia and Eastern Europe studies are limited due to the use of either single-centre data from large tertiary centres or registries that cover only a fraction of the population. There is tremendous heterogeneity with regards to the results of different epidemiology studies from various countries being reported. This makes drawing clear conclusions very difficult and this is compounded by histological classification of NETs and the changes with different WHO iterations and ICD coding.

Another limitation is the lack of healthcare resource and registries in Africa and other developing areas. From the SEER database, it appears that Africans and Hispanics have increased incidence of rectal NETs; however, there is no published data on rectal NET incidence in African countries or South America/Mexico. As to the reported incidence of rectal NETs in Asians, it is likely that this is due to genetic factors in addition to screening practices since the high incidence of rectal NETs is seen both in Asian databases from Korea and Taiwan as well as being replicated in SEER database findings.

## Conclusion

In summary, there appears to be a significant global variation in rectal NETs, which is related in part to clinical practice, availability of colon cancer screening and ethnic variation. The data to support ethnic variability is best demonstrated with data from SEER database which assesses incidence of GEP-NETs in different ethnicities without multiple variables. Yao et al. demonstrated a higher incidence of rectal NET in Asian and African Americans compared to White populations. Conversely, there is a lower incidence of bronchial and small bowel NETs in African Americans compared with White populations.

Global studies have demonstrated an increasing incidence of rectal NETs. A significant factor in the increasing incidence is related to increased use of diagnostic investigations. Primarily, endoscopy is being undertaken for colon cancer screening purposes, and the most common use of endoscopy, specifically colonoscopy, is as a diagnostic tool. As screening programmes for colorectal cancer continue to evolve and encompass more of the population, there would be an expected increasing incidence of rectal NETs; however, this will eventually plateau as reported in some of the SEER data with no longer increasing incidence of rectal NETs in the 60–70 age cohort. The mean age of diagnosis appears to be similar in several studies at around 55–60 years of age. There is evidence of ethnic variation in terms of development of NETs, with rectal NETs being more commonly diagnosed in the Asian, African and Hispanic populations. The genetic drivers for this increased incidence have not been elucidated to date. The ethnic variation seen in the SEER database amongst Asian individuals is replicated in the Asian data series from Taiwan, Korea, Japan and China. Therefore, suggesting this is genetically rather than environmentally related and not reduced by migration to other countries. There may be as of yet undetermined environmental factors leading to increasing incidence of rectal NETs. Standardized data collection methods and population-based registries would enable true understanding of epidemiology of NETs and a better understanding of healthcare utilization. This in turn may enable identification of factors that lead to global variation in incidence of rectal NETs.

## References

[1] Dasari A (2017). Trends in the incidence, prevalence, and survival outcomes in patients with neuroendocrine tumors in the United States. JAMA Oncol.

[2] • Yao JC, et al. One hundred years after ‘carcinoid’: epidemiology of and prognostic factors for neuroendocrine tumors in 35,825 cases in the United States. J Clin Oncol*. *2008;26(18): 3063–3072. **This is a seminal paper that was the first to clearly demonstrate the high prevalance of NETs in the USA and also the increasing incidence**. 10.1200/JCO.2007.15.4377 18565894

[3] Genus TSE (2019). Impact of neuroendocrine morphology on cancer outcomes and stage at diagnosis: a UK nationwide cohort study 2013–2015. Br J Cancer.

[4] Yamaguchi T (2021). A nationwide, multi-institutional collaborative retrospective study of colorectal neuroendocrine tumors in Japan. Ann Gastroenterol Surg.

[5] Zhang X, Ma L, Bao H, Zhang J, Wang Z, Gong P (2014). Clinical, pathological and prognostic characteristics of gastroenteropancreatic neuroendocrine neoplasms in China: a retrospective study. BMC Endocr Disord.

[6] Volante M (2021). Neuroendocrine neoplasms of the appendix, colon and rectum. Pathologica.

[7] Kim JY (2015). Non-L-cell immunophenotype and large tumor size in rectal neuroendocrine tumors are associated with aggressive clinical behavior and worse prognosis. Am J Surg Pathol.

[8] Sohn JH (2015). Prognostic Significance of defining L-cell type on the biologic behavior of rectal neuroendocrine tumors in relation with pathological parameters. Cancer Res Treat.

[9] Ramage JK (2016). ENETS consensus guidelines update for colorectal neuroendocrine neoplasms. Neuroendocrinology.

[10] Basuroy R, Haji A, Ramage JK, Quaglia A, Srirajaskanthan R (2016). Review article: the investigation and management of rectal neuroendocrine tumours. Aliment Pharmacol Ther.

[11] Yoon SN, Yu CS, Shin US, Kim CW, Lim S-B, Kim JC (2010). Clinicopathological characteristics of rectal carcinoids. Int J Colorectal Dis.

[12] Moon CM (2016). Long-term clinical outcomes of rectal neuroendocrine tumors according to the pathologic status after initial endoscopic resection: a KASID multicenter study. Am J Gastroenterol.

[13] Boyar Cetinkaya R, Aagnes B, Thiis-Evensen E, Tretli S, Bergestuen DS, Hansen S (2017). Trends in incidence of neuroendocrine neoplasms in Norway: a report of 16,075 cases from 1993 through 2010. Neuroendocrinology.

[14] • Yu Y-J, Li Y-W, Shi Y, Zhang Z, Zheng M-Y, Zhang S-W. Clinical and pathological characteristics and prognosis of 132 cases of rectal neuroendocrine tumors. World J Gastrointest Oncol. 2020;12(8): 893–902. **The paper demonstrates the importance of histological and clinical charcteristics of rectal NETs**. 10.4251/wjgo.v12.i8.893 PMC7443838 32879666

[15] Son J, Park IJ, Yang DH, Kim J, Kim KJ, Byeon JS, Hong SM, Kim YI, Kim JB, Lim SB, Yu CS, Kim JC. Oncological outcomes according to the treatment modality based on the size of rectal neuroendocrine tumors: a single-center retrospective study. Surg Endosc. 2021. 10.1007/s00464-021-08527-6. 10.1007/s00464-021-08527-6 34009477

[16] Masui T, Ito T, Komoto I, Uemoto S (2020). Recent epidemiology of patients with gastro-entero-pancreatic neuroendocrine neoplasms (GEP-NEN) in Japan: a population-based study. BMC Cancer.

[17] Caplin M (2012). ENETS consensus guidelines for the management of patients with digestive neuroendocrine neoplasms: colorectal neuroendocrine neoplasms. Neuroendocrinology.

[18] • Inada Y, et al*.* Risk of lymph node metastasis after endoscopic treatment for rectal NETs 10 mm or less. Int J Colorectal Dis. 2021;36(3): 559–567. **A small study that highlights the risk of lymph node metastases in patients with small rectal NETs**. 10.1007/s00384-020-03826-1 33388960

[19] Fine C (2019). Endoscopic management of 345 small rectal neuroendocrine tumours: a national study from the French group of endocrine tumours (GTE). United Eur Gastroenterol J.

[20] Konishi T (2007). Prognosis and risk factors of metastasis in colorectal carcinoids: results of a nationwide registry over 15 years. Gut.

[21] Takita M (2019). Clinical Outcomes of patients with small rectal neuroendocrine tumors treated using endoscopic submucosal resection with a ligation device. Digestion.

[22] Ushigome H (2019). Difficulty of predicting lymph node metastasis on CT in patients with rectal neuroendocrine tumors. PLoS One.

[23] Mandair D, Caplin ME (2012). Colonic and rectal NET’s. Best Pract Res Clin Gastroenterol.

[24] Fraenkel M, Kim M, Faggiano A, de Herder WW, Valk GD, Knowledge NETwork (2014). Incidence of gastroenteropancreatic neuroendocrine tumours: a systematic review of the literature. Endocr Relat Cancer.

[25] Ellis L, Shale MJ, Coleman MP (2010). Carcinoid tumors of the gastrointestinal tract: trends in incidence in England since 1971. Am J Gastroenterol.

[26] Das S, Dasari A (2021). Epidemiology, incidence, and prevalence of neuroendocrine neoplasms: are there global differences?. Curr Oncol Rep.

[27] Alwan H (2020). Incidence trends of lung and gastroenteropancreatic neuroendocrine neoplasms in Switzerland. Cancer Med.

[28] Scherübl H (2013). Clinically detected gastroenteropancreatic neuroendocrine tumors are on the rise: epidemiological changes in Germany. World J Gastroenterol.

[29] Lawrence B, Gustafsson BI, Chan A, Svejda B, Kidd M, Modlin IM (2011). The epidemiology of gastroenteropancreatic neuroendocrine tumors. Endocrinol Metab Clin North Am.

[30] Hallet J, Law CHL, Cukier M, Saskin R, Liu N, Singh S (2015). Exploring the rising incidence of neuroendocrine tumors: a population-based analysis of epidemiology, metastatic presentation, and outcomes. Cancer.

[31] Palepu J (2017). Trends in diagnosis of gastroenteropancreatic neuroendocrine tumors (GEP-NETs) in India: a report of multicenter data from a web-based registry. Indian J Gastroenterol.

[32] Ito T, Lee L, Jensenc RT (2018). Carcinoid-syndrome: recent advances, current status and controversies. Curr Opin Endocrinol Diabetes Obes.

[33] Fan J-H (2017). A nation-wide retrospective epidemiological study of gastroenteropancreatic neuroendocrine neoplasms in china. Oncotarget.

[34] Gastrointestinal Pathology Study Group of Korean Society of Pathologists (2012). Current trends of the incidence and pathological diagnosis of gastroenteropancreatic neuroendocrine tumors (GEP-NETs) in Korea 2000–2009: multicenter study. Cancer Res Treat.

[35] Lim C-H (2017). Incidence and clinical characteristics of gastroenteropancreatic neuroendocrine tumor in Korea: a single-center experience. Korean J Intern Med.

[36] Tsai H-J, Wu C-C, Tsai C-R, Lin S-F, Chen L-T, Chang JS (2013). The epidemiology of neuroendocrine tumors in Taiwan: a nation-wide cancer registry-based study. PLoS One.

[37] Huang P-Y (2018). Prognostic factors of patients with gastroenteropancreatic neuroendocrine neoplasms. Kaohsiung J Med Sci.

[38] Chang JS, Chen L-T, Shan Y-S, Chu P-Y, Tsai C-R, Tsai H-J (2021). An updated analysis of the epidemiologic trends of neuroendocrine tumors in Taiwan. Sci Rep.

[39] Chang J, Chen L-T, Shan Y-S, Chu P-Y, Tsai C-R, Tsai H-J. An updated analysis of the epidemiologic trends of neuroendocrine tumors in Taiwan. Scientific Reports. 2021;11. 10.1038/s41598-021-86839-2. 10.1038/s41598-021-86839-2 PMC8041887 33846396

[40] Kaminski M, Polkowski M. Prevalence and endoscopic features of rectal neuroendocrine tumours (carcinoids) among 50148 participants of Polish colorectal-cancer screening programme. Gut. 2007;56:a310.

[41] Kooyker AI, Verbeek WH, Berg JG, Tesselaar ME, Leerdam ME (2020). Change in incidence, characteristics and management of colorectal neuroendocrine tumours in the Netherlands in the last decade. United Eur Gastroenterol J.

[42] Taghavi S, Jayarajan SN, Powers BD, Davey A, Willis AI (2013). Examining rectal carcinoids in the era of screening colonoscopy. Dis Colon Rectum.

[43] Shen C (2019). Racial differences in the incidence and survival of patients with neuroendocrine tumors. Pancreas.

[44] May FP, Glenn BA, Crespi CM, Ponce N, Spiegel BMR, Bastani R (2017). Decreasing Black-White disparities in colorectal cancer incidence and stage at presentation in the United States. Cancer Epidemiol Biomarkers Prev.

